# Selective Anticancer Activity and Safety Profile of Chlorochalcones: Impact on Breast Cancer, Blood, and Endothelial Cells

**DOI:** 10.3390/cells14161299

**Published:** 2025-08-21

**Authors:** Sylwia Cyboran-Mikołajczyk, Karolina Matczak, Teresa Kaźmierczak, Natalia Trochanowska-Pauk, Tomasz Walski, Raghvendra Bohara, Karol Bukowski, Agnieszka Krawczyk-Łebek, Edyta Kostrzewa-Susłow

**Affiliations:** 1 Department of Physics and Biophysics, Faculty of Biotechnology and Food Sciences, Wrocław University of Environmental and Life Sciences, Norwida 25 St., 50-375 Wrocław, Poland; teresa.kazmierczak@upwr.edu.pl (T.K.); natalia.trochanowska-pauk@upwr.edu.pl (N.T.-P.); 2 Department of Medical Biophysics, Faculty of Biology and Environmental Protection, University of Lodz, Pomorska 141/143 St., 90-236 Lodz, Poland; karolina.matczak@biol.uni.lodz.pl (K.M.); karol.bukowski@biol.uni.lodz.pl (K.B.); 3 Department of Biomedical Engineering, Faculty of Fundamental Problems of Technology, Wrocław University of Science and Technology, Wybrzeże Wyspiańskiego 27, 50-370 Wrocław, Poland; tomasz.walski@pwr.edu.pl; 4 Centre for Interdisciplinary Research, D.Y. Patil Educational Society, Kolhapur 416006, India; raghvendrabohara@gmail.com; 5 Department of Food Chemistry and Biocatalysis, Wrocław University of Environmental and Life Sciences, Norwida 25 St., 50-375 Wrocław, Poland; agnieszka.krawczyk-lebek@upwr.edu.pl (A.K.-Ł.); edyta.kostrzewa-suslow@upwr.edu.pl (E.K.-S.)

**Keywords:** RBCs, leukocytes, PLTs, membrane potentials, HMEC-1, H_2_DCFDA, molecular mechanism, biological activity, aggregometry, toxicity, structure activity relationship

## Abstract

In the pursuit of novel anticancer therapies, assessing their selectivity and safety profile towards healthy cells is crucial. This study investigated chlorochalcones, derivatives of 2′-hydroxychalcone containing a chlorine atom, for their impact on human breast cancer cells (MCF-7 and MDA-MB-231), healthy blood cells (erythrocytes, peripheral blood mononuclear cells (PBMCs), platelets), and microvascular endothelial cells (HMEC-1). Our findings demonstrated that chlorochalcones did not detrimentally affect erythrocytes, showing no hemolysis or preserving osmotic resistance and transmembrane potential. They also exhibited minimal impact on normal PBMC viability and varying effects on platelet metabolic activity at therapeutic concentrations. Importantly, these derivatives displayed lower toxicity towards HMEC-1 endothelial cells than towards breast cancer cells, indicating a degree of selectivity. Chlorochalcones have high antiproliferative activity against cancer cells, primarily by inducing apoptosis with virtually no significant impact on cell cycle progression. Their mechanism of action involves the modulation of reactive oxygen species (ROS) levels and induction of mitochondrial dysfunction, including membrane depolarization and reduced mitochondrial mass. Biological activity, including toxicity and ROS modulation, is dependent on the position and number of chlorine atoms. In conclusion, this study highlights the ability of chlorochalcones to effectively target malignant cells while sparing normal circulatory and endothelial cells, thus positioning them as a promising class of candidates for further anticancer drug development.

## 1. Introduction

The pursuit of novel, effective, and safe anticancer therapies is a key challenge in modern medicine and pharmacology. A fundamental limitation of many conventional chemotherapeutic agents, such as taxanes or doxorubicin, is their low selectivity, which leads to systemic toxicity and numerous adverse effects [1]. Consequently, an ideal oncological drug candidate should exhibit not only high efficacy in eliminating cancer cells but a minimal impact on healthy cells and tissues. In this context, chalcones (1,3-diphenyl-2-propen-1-one) a class of compounds belonging to the flavonoid family, have garnered significant interest, as they are distinguished by a broad and well-documented spectrum of biological activity. The scientific literature describes their antibacterial, antifungal, and anti-inflammatory abilities, as well as, most notably, their ability to inhibit the proliferation of various cancer cell lines [2,3,4,5]. Research has indicated that chemical modifications, such as the introduction of a chlorine atom into the chalcone structure, can significantly enhance its biological activity [2,6]. Numerous molecular mechanisms underlying the anticancer action of chalcones have been described, including induction of apoptosis and autophagy, cell cycle arrest, inhibition of angiogenesis, and modulation of key signaling pathways [7,8,9,10].

From a structure–activity relationship perspective, halogenation, particularly with chlorine, modulates the behavior of chalcones by integrating electron-withdrawing effects with physicochemical adjustment of lipophilicity and permeability, which are characteristics that can influence potency and selectivity [6,11]. Within the 2′-hydroxychalcone subfamily, intramolecular hydrogen bonding stabilizes the enone and favors a near-planar conformation, which can influence target engagement [12]. Reports in the literature have indicated that chlorine substitution on the B-ring of chalcones significantly modulates their biological activity in a position-dependent manner. Differences in potency and selectivity profiles have been observed across various cancer models, often associated with modulation of redox homeostasis and mitochondrial dysfunction [13,14,15]. In 2′-hydroxychalcones, chlorine substitution on the A ring has more frequently been associated with reduced antiproliferative, antioxidant, and anti-inflammatory activities compared with B-ring-chlorinated analogs, underscoring the ring- and position-specific nature of structure–activity relationships (SARs) [6,16,17].

In the search for effective anticancer agents, 2′-hydroxychalcones containing a chlorine atom have emerged as promising candidates because of their structural flexibility, which allows for targeted modifications to enhance toxicity against MCF-7 and MDA-MB-231 breast cancer cells, as confirmed by our previous studies [6]. Despite extensive knowledge of the general activity of chalcones, significant research gaps remain. First, the mechanisms of action of specific derivatives, such as 2′-hydroxychalcones containing chlorine, against aggressive cancer types, including triple-negative breast cancer (TNBC), represented by the MDA-MB-231 cell line, have not been fully elucidated. Second, most research has focused on assessing anticancer efficacy while overlooking systematic and comprehensive safety analyses. There is a scarcity of studies that simultaneously evaluate the impact of these compounds on normal cells essential for organismal physiology, such as blood components (erythrocytes, platelets, mononuclear cells) and vascular endothelial cells [10,18,19,20,21]. Such a dual-pronged analysis is essential for thorough assessment of the therapeutic potential and safety profile of new drug candidates.

Given the significant impact of chemotherapy on the circulatory system, evaluating the effects of chlorochalcones on blood, endothelial, and breast cancer cells offers a comprehensive view of their therapeutic potential and safety profile. In light of these considerations, the aim of this study was to evaluate a series of 2′-hydroxychalcone derivatives containing a chlorine atom, assessing both their cytotoxicity against key circulatory system cells and the mechanisms of their action underlying selective anticancer activity. The first objective involved evaluating the impact of the compounds on the structural and functional integrity of erythrocytes, platelets (PLT), peripheral blood mononuclear cells (PBMCs), and human microvascular endothelial cells (HMEC-1). The second key objective was to investigate the mechanism responsible for the antiproliferative action of these compounds against breast cancer cells (MCF-7 and MDA-MB-231), with a particular focus on their ability to induce apoptosis, their impact on the cell cycle, and their effect on mitochondrial function.

## 2. Materials and Methods

### 2.1. Chloroderivatives of 2′-Hydroxychalcone and Chemical Reagents

**Figure 1:**
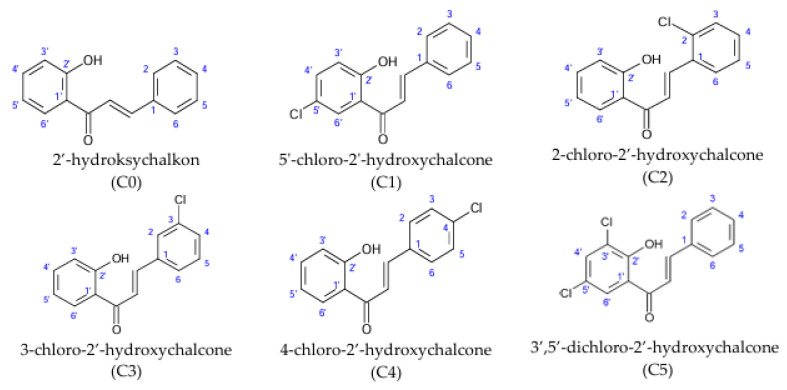
Chemical structures of 2′-hydroxychalcone and its chlorine derivatives.

Chlorine derivatives of 2′-hydroxychalcone (C0), 5′-chloro-2′-hydroxychalcone (C1), 2-chloro-2′-hydroxychalcone (C2), 3-chloro-2′-hydroxychalcone (C3), 4-chloro-2′-hydroxychalcone (C4), and 3′,5′-dichloro-2′-hydroxychalcone (C5) were obtained via the Claisen–Schmidt condensation reaction. The chemical synthesis of chlorine derivatives of 2′-hydroxychalcone and physical data (color and form, molecular ion mass, molecular formula, melting point (°C), retention time tR (min), retardation factor Rf, and NMR spectral data) of the obtained compounds were published previously [6]. The chemical structures are presented in Figure 1.

Chemicals used for the preparation of the buffers were as follows: NaCl, KH_2_PO_4_ (Avantor Performance Materials, Gliwice, Poland), Na_2_HPO_4_·12H_2_O, NaH_2_PO_4_·H_2_O, sodium-EDTA, Tris (Chempur, Piekary Śląskie, Poland), KCl, NaOH (STANLAB Sp. z o. o., Lublin, Poland), NH_4_Cl, and NaHCO_3_ (EUROCHEM BGD Sp. z o. o., Tarnów, Poland). Glutaraldehyde, dimethyl sulfoxide (DMSO), immersion oil, 1-propanol, Lymphosep, RPMI-1640, Hank’s Balanced Salt Solution, Dulbecco’s modified Eagle’s medium (DMEM), MCBD-131, phosphate-buffered saline, 3,3′-dipropylthiadicarbocyanine iodide (DiSC_3_(5),4-[4-[(1E,3E)-4-[4-(dipentylamino)phnyl]buta-1,3-dienyl]pyridin-1-ium-1-yl]butane-1-sulfonate (RH421), 2′,7′-dichlorodihydrofluorescein diacetate (H_2_DCF-DA), and monodansylcadaverine (MDC) were purchased from Merck (Dramstadt, Germany). CaCl_2_ was purchased from Chempur (Piekary Śląskie, Poland). Methanol and ethanol were purchased from STANLAB Sp. z o. o. (Lublin, Poland). Collagen was obtained from Chrono-Log (Havertown, PA, USA). Tetrazolium chloride (XTT), phenazine methasulfate (PMS), MitoTracker Red FM, MitoTracker Green FM, fetal bovine serum (FBS), penicillin/streptomycin, and an Annexin V–FITC/PI apoptosis kit were obtained from ThermoFisher Scientific (Waltham, MA, USA).

### 2.2. Human Blood Cells

Peripheral blood and platelet-rich plasma were purchased from the Regional Center of Blood Donation and Hemotherapy, Prof. Dr. Tadeusz Dorobisz, Wroclaw, under contract with Wroclaw University of Environmental and Life Sciences. This research did not require ethics committee approval under Polish law, as blood samples were anonymized and consisted of undercaps and samples remaining after red blood cell concentrate extraction. Erythrocytes and peripheral blood mononuclear cells (PBMCs) were extracted from peripheral blood, whereas thrombocytes were sourced from platelet-rich plasma through apheresis. An isotonic phosphate solution with pH 7.4 (131 mM NaCl, 1.79 mM KCl, 0.86 mM MgCl_2_, 11.79 mM Na_2_HPO_4_·2H_2_O, 1.80 mM Na_2_H_2_PO_4_ˑH_2_O) was used to cleanse erythrocytes. Mononuclear cells (PBMCs) were isolated by density gradient centrifugation as outlined by Kluska et al. [22]. The leukocyte layer was combined with PBS-EDTA at 1:4 ratio, treated with erythrocyte lysis buffer, and rinsed with PBS. Cells were suspended in RPMI 1640 medium containing 10% fetal bovine serum (FBS), 1 µg/mL hydrocortisone, and 1% penicillin/streptomycin from Gibco and Sigma Aldrich (Waltham, MA, USA and St. Louis, MO, USA) and incubated for 24 h at 37 °C with 5% CO_2_ (MIDI 40 CO_2_ Incubator, Thermo Fisher Scientific, Waltham, MA, USA).

### 2.3. HMEC-1, MCF-7, and MDA-MB-231 Cell Cultures

In this study, MCF-7 and MDA-MB-231 breast cancer cells served as representative models of neoplastic cells. The normal cellular counterparts were exemplified by HMEC-1, an immortalized human microvascular endothelial cell line. All the cell lines were obtained from ATCC (Rockville, MD, USA).

The cancer cell lines were propagated as monolayers in Dulbecco’s modified Eagle’s medium (DMEM), which was enriched with 10% (*v*/*v*) fetal bovine serum (FBS) and 1% (*v*/*v*) penicillin/streptomycin solution (containing 10,000 U/mL penicillin and 10 mg/mL streptomycin). Cultivation was carried out in a humidified atmosphere at 37 °C with 5% CO_2_. Endothelial cells (HMEC-1) were cultured under similar environmental conditions (37 °C, humidified atmosphere, 5% CO_2_) using MCDB 131 medium supplemented (Gibco, Waltham, MA, USA) with 10% FBS (Sigma-Aldrich, St. Louis, MO, USA), 10 ng/mL epidermal growth factor (EGF) (Thermo Fisher Scientific, Waltham, MA, USA), 1 µg/mL hydrocortisone (Sigma-Aldrich, St. Louis, MO, USA), and the aforementioned penicillin/streptomycin solution. All cell lines were used at passages below 30 to minimize phenotypic drift and ensure reproducibility of results.

### 2.4. Spectrophotometric Study of the Degree of Hemolysis and Osmotic Resistance of RBCs

The hemolytic activity of 2′-hydroxychalcone and its chlorine-containing derivatives, as well as their effect on the osmotic resistance of erythrocytes, were assessed using the procedures previously described by Cyboran-Mikołajczyk et al. [23]. Chalcones were dissolved in DMSO and tested at concentrations of 50–250 μM for hemolytic assays and 40 and 100 μM for osmotic resistance evaluation.

### 2.5. Impact of Chlorochalcones on the Transmembrane Potential of RBCs

The transmembrane potential of erythrocytes was assessed using the fluorescence indicator DiSC_3_(5), following the method outlined by Zavodnik et al. [24] with slight alterations. A 5% hematocrit (Hct) erythrocyte solution was incubated with 100 µM chalcone for 1 h at 37 °C. Subsequently, 2 mL of 310 mOsm PBS was added to the mixture, which was then centrifuged for 15 min at room temperature, and the supernatant was discarded to separate the cells from the chalcone solution. Subsequently, 100 µL samples were resuspended in 2.5 mL of buffered saline containing 10 mM Tris-HCl (pH 7.4) and 150 mM KCl + NaCl, with K^+^ concentrations ranging from 10 to 140 mM. An ethanolic solution of DiSC_3_(5) was then added at a final concentration of 2 µM, and the samples were incubated in the dark at room temperature for 45 min. The fluorescence intensity of the dye (IF) was recorded using a fluorimeter (CARRY Eclipse, Varian, Palo Alto, CA, USA) at 660 nm with excitation at 625 nm. Valinomycin, an ionophore, was added to the samples to reach a final concentration of 1 µM. After a 10-min incubation, the fluorescence intensity of the probe (I_v_) was measured again. By plotting the relationship between (I − I_v_)/I and log_2_ of the external K^+^ concentration, the external potassium concentration (K^+^) was determined where no change in DiSC_3_(5) fluorescence intensity was observed upon addition of valinomycin. The transmembrane potential (∆Ψ) was calculated using the Nernst equation for monovalent ions.

### 2.6. Microscopic Studies of Erythrocyte Shapes

Erythrocytes were washed four times with saline solution, resuspended in the same solution, and adjusted to the appropriate chalcone concentrations. The hematocrit of erythrocytes in the modification solution was set to 1.2%, with modifications occurring for 1 h at 37 °C. After modification, the erythrocytes were fixed using 0.2% glutaraldehyde solution. Red blood cells were examined using a Nikon Eclipse E200 biological microscope (Nikon Corporation, Tokyo, Japan) equipped with a digital camera MOTICAM S6 (MoticEurope, S.L.U., Barcelona, Spain). Photomicrographs enabled the counting of erythrocytes with different morphologies. The proportions of the three basic forms (discocytes, echinocytes, and stomatocytes) were quantified in approximately 300 cells. Chalcone concentrations used were 50 and 100 µM. Erythrocyte morphologies were assigned indices according to the Bessis scale [25].

### 2.7. Impact of Chlorochalcones on the Mitochondrial Reductive Capacity of PMBC and Platelets

PBMCs were diluted in RPMI 1640 with 10% FBS containing 2 mM glutamine, 100 μg/mL streptomycin, and 100 UI/mL penicillin and were counted using a Bürker chamber. A 96-well plate was filled with 50 µL of cell suspension at a density of 1 × 10^4^ cells/well. Then, 50 µL of chalcones diluted in RPMI-1640 medium (Gibco, Waltham, MA, USA) was added to the cells to a final concentration in the range of 50–150 µM and incubated for 90 min and 24 h. For platelets collected by apheresis, cells were centrifuged from plasma and resuspended in RPMI-1640 medium. Cell numbers were determined using a BC-2800 Vet hematology analyzer (Mindray Bio-Medical Electronics Co., Ltd., Shenzhen, China) and seeded in 96-well plates at a density of 1 × 10^7^ cells/well. The cells were then treated with chalcones diluted in the same medium to a final concentration of 50–150 µM and incubated for 90 min at 37 °C with 5% CO_2_. Following incubation, 25 µL of XTT/PMS solution (10 µL of 10 mM PMS + 4 mL of 4 mg/mL XTT solution) was added to the cells. Incubation times with XTT/PMS were 4 h for PMBC and 24 h for platelets at 37 °C with 5% CO_2_ in the dark. The absorbance of XTT/PMS-platelets and PBMC containing samples was measured using a microplate spectrophotometer (Epoch, BioTek, Agilent Technologies, Winooski, VT, USA) at wavelengths of 450 and 690 nm.

In the aggregation assay, platelets obtained via apheresis and preserved in plasma were quantified using a BC-2800 Vet hematology analyzer (Mindray Bio-Medical Electronics Co., Ltd., Shenzhen, China) and adjusted to 5 × 10^8^ platelets/mL. Then, 500 µL of platelets was incubated with varying concentrations of chalcones for 30 min at 37 °C. After incubation, the cell count was determined using a hematology analyzer and transferred to a measurement tube. To activate platelets, 25 µL of calcium chloride (25 mM) was added. After 3 min, platelet aggregation was induced by adding 1 µL collagen (1 mg/mL). The platelet suspension was stirred continuously at 1200 rpm. Platelet aggregation was assessed using a Chrono-log 700 light-transmission platelet aggregometer (Chrono-log Corporation, Havertown, PA, USA). The transmission before agonist addition was set as 0% aggregation, whereas transmission after agonist addition to control cells was set as 100% aggregation. The degree of aggregation was determined by the maximum increase in light transmission within 15 min of addition of the inducer. To minimize solvent effects, DMSO concentration in the platelet suspension was maintained below 0.5%.

### 2.8. Inhibitory Effects on HMEC-1 Cell Proliferation

A spectrophotometric assay employing XTT reagent was used to evaluate the antiproliferative effects of the compounds on HMEC-1 cells. Cells in the exponential growth phase were seeded in a 96-well plate at a density of 5 × 10^3^ cells per well. Following a 24-h incubation period, the cells were treated with varying concentrations of chalcones and incubated for an additional 72 h. After incubation, 50 µL of an XTT/PMS solution (comprising 10 µL of 10 mM PMS and 4 mL of 4 mg/mL XTT solution) was added to the cells. The medium was aspirated and removed. The cells were then incubated at 37 °C with 5% CO_2_ in the dark. Following this incubation, the absorbance of the samples was measured at 450 nm and 690 nm using PowerWave HT (BioTek Instruments, Inc., Winooski, VT, USA). In the viability assays, the IC_50_ value, representing the concentration of chalcone required to reduce cell viability by 50% compared with untreated cells, was employed to quantify the cytotoxicity of the compounds under investigation.

### 2.9. Evaluation of the Intensity of the Autophagy Process

Cells were seeded into sterile black 96-well microplates at a density of 5 × 10^3^ cells/well. The compounds were introduced into the cells at appropriate concentrations and incubated for durations ranging from 24 to 72 h. Following incubation, the medium was aspirated, and 100 µL of 50 µM monodansylcadaverine solution was dispensed into each well, followed by incubation in the dark for 15 min at 37 °C. Subsequently, the probe was removed from the wells, and the cells were lysed using a solution containing 10 mM Tris-HCl/0.1% Triton X-100 (pH 8.0). Fluorescence measurements were acquired at an excitation wavelength (λ_ex_) of 390 nm and an emission wavelength (λ_em_) of 460 nm using a Fluoroscan Ascent FL microplate reader (LabSystem, Vantaa, Finland). For standardization purposes, 50 µL of 2 mM ethidium bromide solution was added to each well. The fluorescence of ethidium bromide was measured at an excitation wavelength (λ_ex_) of 530 nm and an emission wavelength (λ_em_) of 590 nm. The results were computed as the ratio of monodansylcadaverine fluorescence to ethidium bromide fluorescence.

### 2.10. Induction of Apoptosis Assessed Through Phosphatidylserine Externalization

The transbilayer movement of phospholipids, specifically phosphatidylserine, was assessed using an Annexin V–FITC staining kit (Invitrogen, Carlsbad, CA, USA). Following experimental procedures, cell pellets were washed twice with PBS (135 mM NaCl, 9 mM Na_2_HPO_4_, 1.5 mM KH_2_PO_4_, 2.7 mM KCl) and subsequently resuspended in 0.5 mL of Annexin V–FITC binding buffer (Invitrogen, Waltham, MA, USA). Prior to analysis, 5 µL of Annexin V–FITC and propidium iodide (PI) were added to the cell suspension. The mixture was then incubated for 5 min in the dark at room temperature. A minimum of 10,000 cells per sample were analyzed using a BD LSR II flow cytometer (Becton Dickinson, San Jose, CA, USA). Based on their distinct staining patterns, cells were categorized as follows: viable cells (Annexin-V-negative and PI-negative), early apoptotic cells (Annexin-V-positive and PI-negative), late apoptotic cells (Annexin-V-positive and PI-positive), and necrotic cells (Annexin-V-negative and PI-positive). The results are presented as the average percentage of cells within each category.

### 2.11. Evaluation of Cell Cycle Phase Distribution

Cells were seeded in 60 mm dishes at a density of 1 × 10^6^ cells per dish in 4 mL of DMEM supplemented with 10% FBS and 1% penicillin/streptomycin solution (10,000 U/mL penicillin and 10 mg/mL streptomycin). The dishes were incubated for 24 h at 37 °C in a humidified atmosphere containing 5% CO_2_. Following this incubation period, the medium was replaced with fresh DMEM-containing test compounds. After 24 or 48 h of treatment, the cells were detached using 0.25% trypsin with 1 mM EDTA, centrifuged at 1000× *g* for 5 min at 4 °C, and resuspended in 100 µL ice-cold PBS (pH 7.4). Fixation was achieved by adding 1 mL chilled 70% ethanol. The fixed cells were centrifuged at 2000× *g* for 10 min at 4 °C to remove ethanol, washed with PBS, and centrifuged again under the same conditions. The resulting pellet was resuspended in 300 µL PBS containing 4 mg/mL propidium iodide and 20 µg/mL RNase A. The cell cycle was assessed using a BD LSR II flow cytometer (Becton Dickinson, San Jose, CA, USA) by analyzing 10,000 events per sample to determine the distribution of cells across different cell cycle phases. Data analysis was conducted using FlowJo v7.6 software (FlowJo, LLC, Ashland, OR, USA).

### 2.12. Assessment of Mitochondrial Membrane Potential and Mass

To evaluate alterations in mitochondrial membrane potential (ΔΨ_m_), MitoTracker Red FM fluorescent lipophilic cationic dye was used. To assess mitochondrial mass, MitoTracker Green FM fluorescent dye was used, which binds to mitochondrial membranes regardless of MMP, enabling the evaluation of mitochondrial content in treated cells. Comparing mitochondrial membrane potential with mass allows differentiation between functional changes and alterations in mitochondrial quantity [26]. Cells were seeded in black 96-well microplates at 5 × 10^3^ cells per well and incubated with test compounds at specified concentrations. After 2 h, the cells were incubated with a potential-dependent probe at 0.1 µM of 30 min. Simultaneously, the cells were stained with a mitochondrial mass probe under identical conditions. Following incubation, the staining solution was removed, and wells were washed with PBS to eliminate excess dye. Fluorescence measurements were conducted using a Fluoroscan Ascent FL microplate reader (LabSystem, Vantaa, Finland). The excitation and emission wavelengths were set at λ_ex_ = 579 nm and λ_em_ = 599 nm for the membrane potential probe and λ_ex_ = 490 nm and λ_em_ = 516 nm for the mitochondrial mass probe.

### 2.13. Intracellular Reactive Oxygen Species (ROS) Measurement

Intracellular reactive oxygen species (ROS) levels were quantified using the fluorescent probe H_2_DCF-DA, a chemically reduced derivative of fluorescein, as an indicator of ROS within cells. Upon cellular entry, the probe undergoes deacetylation by cellular esterases and subsequent oxidation, transforming nonfluorescent H_2_DCF-DA into highly fluorescent 2′,7′-dichlorofluorescein (DCF) [27]. Cells were seeded in black 96-well microplates at a density of 5 × 10^3^ cells per well and exposed to the respective compounds at the specified concentrations. Following incubation, the medium was removed, and 50 µL of the probe in HBSS was added to achieve a final concentration of 5 μM. The cells were then incubated in the dark at 37 °C for 30 min. The fluorescence of DCF was measured at excitation and emission wavelengths of λ_ex_ = 490 and λ_em_ = 529 nm using a Fluoroscan Ascent FL microplate reader (Labsystem, Vantaa, Finland).

### 2.14. Statistical Analysis of the Results

Statistical analyses were conducted using the Statistica 12 software (StartSoft, Inc., Tulsa, OK, USA). Dunnett’s test was primarily applied to compare modified groups with the control group, whereas Tukey’s test was employed for all pairwise comparisons. The significance levels were set at α = 0.05 and α = 0.01 contingently on the specific experiment.

## 3. Results

### 3.1. Interaction of Chlorine Containing Derivatives of 2′-Hydroxychalcone with Blood Cells

**Figure 2:**
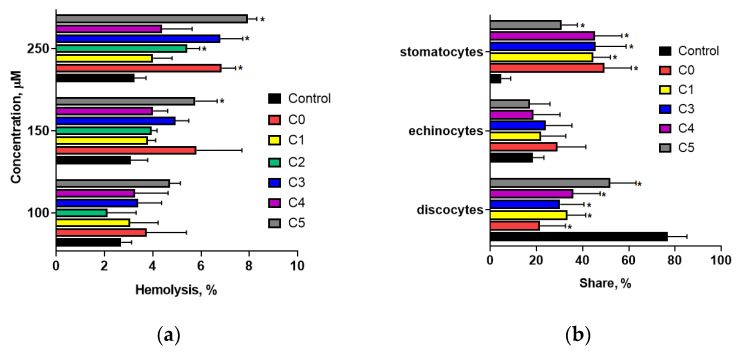
(**a**) Percentage of hemolysis in red blood cells (RBCs) and RBCs treated with the tested compounds at concentrations ranging from 100 to 250 µM. (**b**) Percentage distribution of discocytes, echinocytes, and stomatocytes in control erythrocytes and erythrocytes treated with 100 µM chalcone. Statistically significant differences between control and chalcone-treated erythrocytes are indicated as * *p* < 0.01.

To assess the hemolytic activity of 2′-hydroxychalcone and its chlorinated derivatives, their capacity to induce erythrocyte hemolysis was evaluated. Figure 2a illustrates the percentage of hemolysis in erythrocytes treated for 90 min with the test compounds at concentrations of 100, 150, and 250 µM. The findings revealed no significant increase in red blood cell (RBC) hemolysis when exposed to compounds at concentrations up to 150 µM compared with control cells. This indicated that these compounds did not exert a destructive effect on RBCs within this concentration range. At 250 µM, a slight increase in blood cell hemolysis (not exceeding 8%) was observed for compounds C1, C2, C4, and C5. Considering that the physiological level of erythrocyte hemolysis is approximately 5%, it can be concluded that the tested compounds exhibited negligible hemolytic activity, even at elevated concentrations.

Figure S1a,b (Supplementary Materials) illustrates representative hemolytic curves of erythrocytes treated with 40 µM and 100 µM concentrations of compounds C0, C3, and C5. The C_50_ concentrations, which denote the points at which 50% hemolysis of control and chalcone-treated erythrocytes were observed, are detailed in Table 1. A higher sodium chloride concentration than that of the control cells indicated reduced osmotic resistance of erythrocytes, and vice versa. These findings suggested that the compounds tested at concentrations of 40 µM and 100 µM did not alter the osmotic resistance of erythrocytes, with the exception of compound C3. When applied at a concentration of 100 µM, compound C3 induced a slight shift in the hemolytic curve towards lower NaCl concentrations (Figure S1, Table 1), thereby marginally enhancing the osmotic resistance of erythrocytes.

At a concentration of 40 µM, 2′-hydroxychalcone and its chlorine-containing derivatives did not cause any changes in erythrocyte morphology. However, at 100 µM, these compounds interacted with the erythrocyte membrane, resulting in morphological changes that were primarily characterized by stomatocyte formation (Figure 2b). Example images of blood cells captured using an optical microscope are included in the Supplementary Materials (Figure S2). Among these, two predominant shapes were observed: a shelved cup shape (stomatocyte I) and a cup shape with pronounced invagination (stomatocyte II) [25]. No statistically significant differences in stomatocyte formation were noted between compound C0 and its chlorine-containing derivatives. Only compound C5, possessing two chlorine atoms in the A ring, showed a slight reduction in stomatocyte formation. These findings suggest that a single chlorine atom substitution in the 2′-hydroxychalcone structure does not significantly alter its capacity to bind to the erythrocyte membrane. Conversely, incorporating two chlorine atoms in the A ring partially attenuates the ability of 2′-hydroxychalcone to induce stomatocyte formation.

The effect of the tested compounds on the transmembrane potential of RBCs was evaluated using a dual radiometric method with a DiSC_3_(5) probe. Studies have shown that compounds used at 100 µM do not alter the transmembrane potential of RBCs (Table 1). No statistically significant differences were observed between the transmembrane potential values of control and chalcone-modified RBCs. This suggests that binding of the compounds to the RBC membrane does not affect ion permeation across the erythrocyte membrane.

**Figure 3:**
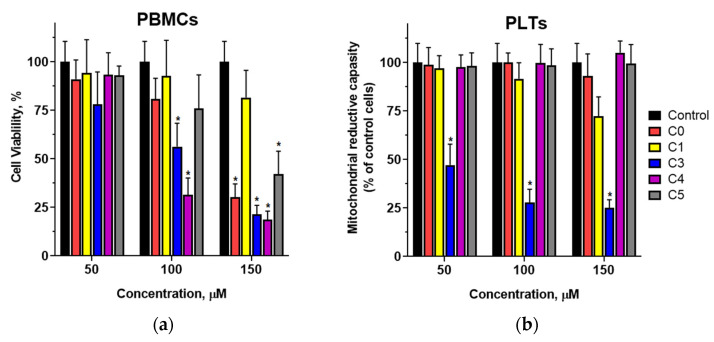
(**a**) Viability of human peripheral blood mononuclear cells (PBMCs) and (**b**) mitochondrial reductive capacity of platelets (PLTs) following treatment with chalcones for 24 h (PBMCs) and 90 min (PLTs), as measured by the XTT assay. PLT results are presented as the percentage of mitochondrial activity relative to that of control cells. Chalcones C0–C5 were tested at concentrations ranging from 50 µM to 150 µM. Statistically significant differences between the control and chalcone-modified cells are expressed as * *p* < 0.01.

The mitochondrial reductive capacity of human peripheral blood mononuclear cells (PBMCs) and platelets was assessed using an XTT assay. Experiments demonstrated that a 24-h incubation of PBMCs with chalcones led to a concentration-dependent decrease in cell viability (Figure 3a), particularly at concentrations of 100 µM and above. At 100 µM, a significant reduction was observed only for compounds C3 and C4, both of which contain a chlorine atom in the B-ring. In contrast, the parent compound (C0) and derivatives with chlorine substitutions in the A-ring (C1 and C5) did not induce changes in the mitochondrial activity of PBMCs at this concentration. Pronounced reductions in PBMC viability were noted for C0, C3, C4, and C5 at 150 µM. The decreases induced by compounds C3 and C4 were slightly more pronounced than those caused by their nonchlorinated counterparts, suggesting that the substitution of a chlorine atom in the B-ring enhances the inhibitory effect of 2′-hydroxychalcone on PBMC mitochondrial activity.

In platelets treated with chalcones at concentrations between 50 and 150 µM for 90 min, no significant effect on cellular metabolic activity was observed (Figure 3b) for C0, C4, or C5. The exceptions were compound C3, which markedly reduced the number of metabolically active cells at concentrations of 50 µM and above, and compound C1 at 150 µM. Moreover, the observed differences in activity for compounds C0, C3, and C4 suggest that both the presence of a chlorine atom and its specific substitution at the 3′ position of the B-ring significantly influence the biological effects of these compounds on platelets.

**Figure 4:**
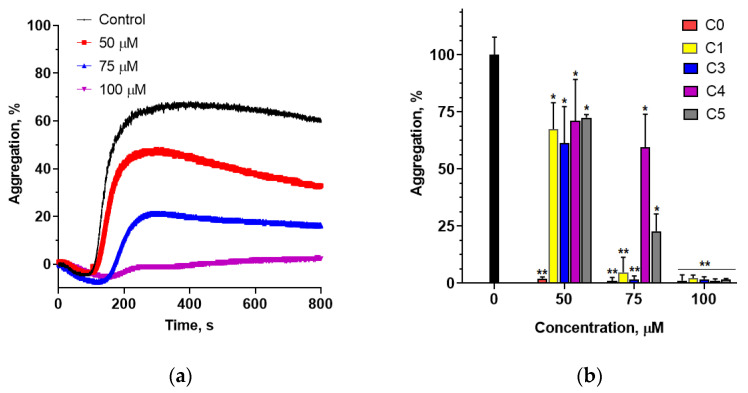
(**a**) Time-dependent changes in platelet aggregation induced by collagen in control platelets (PLTs) and those treated with compound C5 at concentrations ranging from 50 µM to 100 µM. (**b**) Percentage of collagen-induced aggregation of chalcone-modified platelets. Chalcones C0–C5 were tested at concentrations ranging from 50 µM to 100 µM. Statistically significant differences between the control and chalcone-modified cells are expressed as follows: * *p* < 0.05, ** *p* < 0.01.

The ability of chalcones to inhibit collagen-induced platelet aggregation was investigated by optical aggregometry. Platelets were incubated with various concentrations of chalcones for 30 min before activation and aggregation was induced. A representative example of time-dependent inhibition of platelet aggregation by different concentrations of C5 is shown in Figure 4a. The study found that 2′-hydroxychalcone (C0) exhibited the strongest inhibitory effect, completely preventing collagen-induced platelet aggregation at a concentration of 50 µM (Figure 4b). The introduction of a chlorine atom in either the A or B ring significantly diminished the antiaggregatory effect of 2′-hydroxychalcone. Among the chlorine-containing derivatives, C1 and C3 demonstrated the highest inhibition of platelet aggregation. A comparison of compounds C3 and C4, both containing a chlorine atom in the B ring, indicated that the position of chlorine substitution in the B ring plays a crucial role in modulating the activity. Moreover, all the tested derivatives completely inhibited collagen-induced platelet aggregation at a concentration of 100 µM.

### 3.2. Inhibitory Effects of Chlorochalcones on HMEC-1 Cell Proliferation

The XTT assay was used to determine the antiproliferative activity of the chlorine-containing chalcones. The concentrations of the compounds (IC_50_) responsible for 50% inhibition of HMEC-1 after 72 h of their modification are provided in Table S1. The IC_50_ values for compounds C0, C1, C2, C3, C4, and C5 are 17.9 ± 0.5, 51.5 ± 2.6, 16.8 ± 0.4, 63.9 ± 2.2, 15.3 ± 0.7 and 38.3 ± 0.9 M, respectively. The results indicated that the chalcones inhibited endothelial cell growth to varying degrees. Derivatives containing a chlorine atom in the A ring, that is, C1 and C5, showed lower inhibitory activity than 2′-hydroxychalcone (C0). In the case of compounds possessing chlorine in the B ring (C2–C4), the antiproliferative activity was not significantly different from that of their nonchlorinated counterpart. The exception was compound C3, which showed significantly reduced ability to inhibit cell growth. These findings suggest that the substitution site of the chlorine atom in the B ring significantly affects the antiproliferative activity of these compounds in HMEC-1 cells.

To determine whether the observed antiproliferative effects of chalcones were selective for endothelial cells, we compared the half-maximal inhibitory concentrations (IC_50_ values) determined for HMEC-1 cells with those obtained for the MDA-MB-231 and MCF-7 cell lines (results are presented in Table S1 and were published earlier in [6]). C0 demonstrated moderate selectivity for HMEC-1 (IC_50_ ratio: 3.4–4.2), whereas C1, C3, and C5 exhibited the highest cytotoxicity towards breast cancer cell lines. Furthermore, IC_50_ values for the inhibition of HMEC-1 and MDA-MB-231 proliferation obtained with C2 and C4 were similar but almost twofold weaker than those obtained for MCF-7 cells. Interestingly, the results showed that in the case of vascular endothelial cells, the antiproliferative activity of 2′-hydroxychalcone remained unchanged or decreased after substitution with a chlorine atom, in contrast to cancer cells.

### 3.3. The Mechanism of the Antiproliferative Action of Chlorochalcones on Breast Cancer Cells

**Figure 5:**
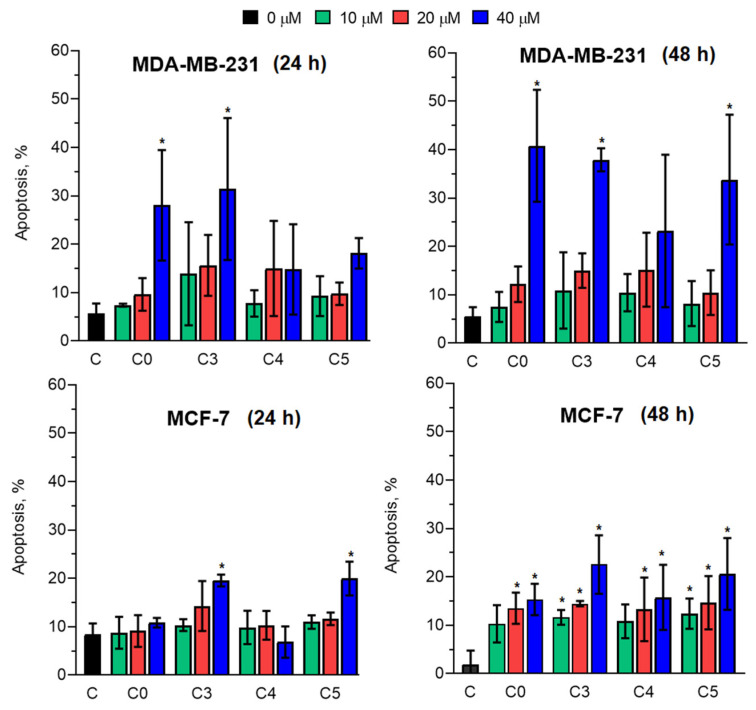
The percentage of apoptosis in control (C) and chalcone-treated MCF-7 and MDA-MB-231 breast cancer cells, as assayed by flow cytometry. Cells were incubated for 24 or 48 h with chalcones C0–C5 at concentrations ranging from 10–40 µM. Statistically significant differences between control and chalcone-treated cells are expressed as * *p* < 0.05.

The ability of the compounds to induce apoptosis was determined by flow cytometry. The cells were treated with the compounds for 24 and 48 h and stained with Annexin V–FITC and PI. Representative Annexin V–FITC/propidium iodide dot plots illustrating apoptosis in MCF-7 and MDA-MB-231 cells after 24 h and 48 h of exposure to chlorochalcones are shown in Figure S3 (Supplementary Materials). The average percentage of apoptotic cells is shown in Figure 5.

The results indicated that the tested compounds possessed varying capacities to induce apoptosis in breast cancer cells. After 24 h of incubation, a twofold increase in the number of apoptotic cells compared with control cells was observed for compounds C3 and C5 (at 40 µM) in the MCF-7 cell population and a threefold increase was observed for compounds C0 and C3 in the MDA-MB-231 cells. Extending the incubation time to 48 h revealed that all tested compounds, when used at concentrations of 20 µM and 40 µM, induced apoptosis in MCF-7 cells. For the second cell line tested, compounds C0, C3, and C5 exhibited significant apoptotic activity, although only at the highest concentration.

**Figure 6:**
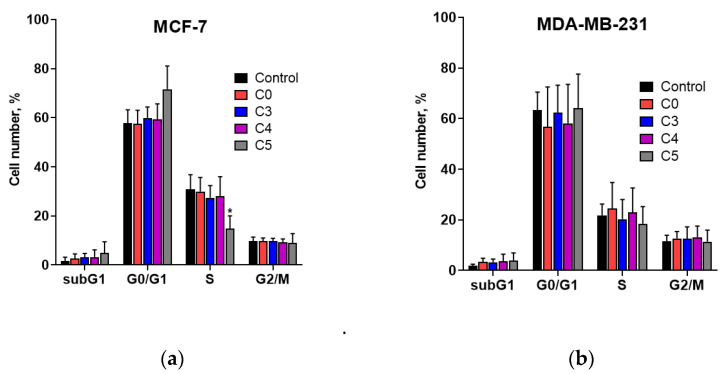
Distribution of MCF-7 (**a**) and MDA-MB-231 (**b**) cells in different phases of the cell cycle after treatment with 40 µM chalcones relative to the untreated control cells. Each graph presents the percentage ± SD of cells in each phase of the cell cycle (sub G1, G0/G1, S, G2/M) obtained from three independent experiments. Statistically important results are expressed as * *p* < 0.05.

Next, the ability of chlorochalcones to modulate the cell cycle was investigated. Representative cell-cycle histograms of propidium-iodide-stained MCF-7 and MDA-MB-231 cells collected 24 h after treatment with chlorochalcones are shown in Figure S4 (Supplementary Materials). The results presented in Figure 6a,b indicate that the chalcones tested had a negligible effect on the cell cycle progression of MCF-7 and MDA-MB-231 cells after 48 h of treatment. The sole exception was compound C5, which induced a slight increase in MCF-7 cell population within the G0/G1 phase and a corresponding decrease in the S phase, suggesting that C5 may arrest cells in the G0/G1 phase and thereby inhibit their entry into the S phase.

**Figure 7:**
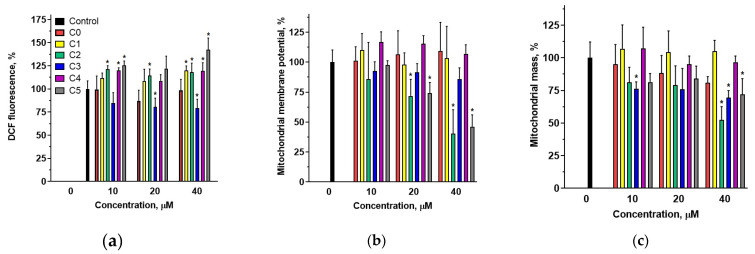
Percentage change in intracellular ROS level (**a**), mitochondrial membrane potential (**b**), and mitochondrial content (**c**) in MDA-MB-231 cells untreated (0) or treated with chalcones C0–C5 at concentrations ranging from 10 to 40 µM. Statistical significance was set at * *p* < 0.05.

The effect of chalcones on ROS levels in MDA-MB-231 cells was determined fluorometrically using the H_2_DCF-DA probe. The results indicated that, within the concentration range of 10–40 µM, 2′-hydroxychalcone did not induce significant alterations in ROS levels compared with control cells (Figure 7a). In contrast, an increase in intracellular ROS was observed for compounds C2 and C5 across the entire concentration range and for compounds C1 and C4 at the highest concentration. Interestingly, in the case of compound C3, which, similarly to compounds C2 and C4, possessed a chlorine atom in the B ring, a slight decrease in ROS levels was observed across the tested concentration range.

The ability of chalcones to modify the mitochondrial membrane potential was determined using the fluorescence probe MitoTracker Red FM. The accumulation of this probe is dependent on membrane potential and does not label dysfunctional mitochondria or those with an altered membrane potential. Mitochondrial depolarization induced by C3 and C5 treatments (at 20 and 40 µM) decreased the fluorescence intensity of MitoTracker Red (Figure 7b). In contrast, other compounds did not affect the fluorescence of the probe, indicating that they did not modify the mitochondrial membrane potential within the tested concentration range.

Alterations in mitochondrial mass induced by the studied compounds were evaluated using the fluorescence probe MitoTracker Green FM (MTG, Thermo Fisher Scientific). MDA-MB-231 cells stained with this probe showed a reduction in fluorescence intensity after treatment with C0, C2, C3, and C5 at a concentration of 40 µM (Figure 7c). The most significant decrease in fluorescence intensity occurred for compounds C2 and C3, both possessing a chlorine atom in the B ring. Conversely, C4, despite containing a chlorine atom in the B ring, did not affect the fluorescence of the probe. This observed reduction in fluorescence intensity signified a decrease in the mitochondrial content.

**Figure 8:**
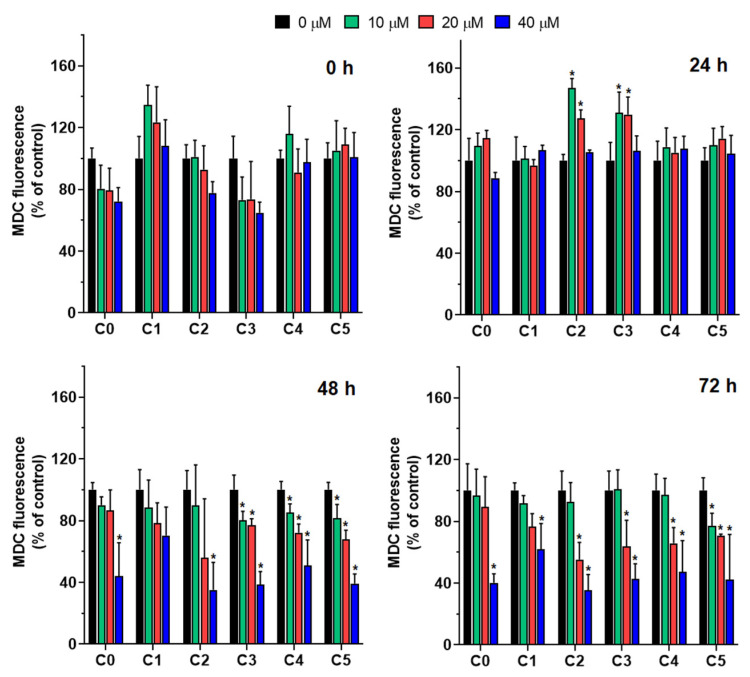
MDC fluorescence in MDA-MB-231 breast cancer cells treated with C0–C5 to assess autophagic activity. Fluorescence was measured at four time points (0 h, 24 h, 48 h, and 72 h) at 10, 20, and 40 µM. MDC levels are expressed as a percentage relative to untreated control cells (C0). Statistically significant differences between control and compound-treated cells are indicated by * *p* < 0.05.

MDC fluorescence was used to assess autophagic activity in MDA-MB-231 cells treated with compounds C1–C5 at concentrations of 10, 20, and 40 µM over time (Figure 8). Monodansylcadaverine (MDC), a fluorescent compound with a dansyl moiety conjugated with cadaverine, preferentially accumulates in autophagosomes via ion trapping and interactions with membrane lipids.

At the initial time point (0 h), no significant differences in MDC levels were observed between the treated cells and the control cells, irrespective of the compound type or concentration. This suggests a lack of immediate autophagic response to compound exposure. However, after 24 h, an increase in MDC content was observed, particularly in cells treated with C2 and C3 at 10 and 20 µM. This increase indicated the possible early activation of autophagy by these agents. As the incubation period was extended to 48 h, the MDC content began to decline notably in cells treated with higher concentrations (20 and 40 µM), especially those exposed to C3, C4, and C5. This reduction suggests a transition in cellular response, potentially reflecting autophagic flux or cytotoxic effects. At 10 µM, MDC levels remained relatively stable, with compounds C3, C4, and C5 exhibiting minimal changes, highlighting a dose-dependent effect. After 72 h, MDC levels significantly decreased across all treatment groups, most prominently in cells treated with C2 and C4 at 20 and 40 µM, respectively. These compounds consistently induced the strongest reduction in MDC fluorescence, with levels dropping below 50% of the control. The observed decline over time suggests a sustained effect of these compounds on autophagic activity, which may result from prolonged autophagic induction or cellular damage.

Overall, the data revealed time- and concentration-dependent effects of the tested compounds on MDC fluorescence. Among these compounds, C3, C4, and C5 consistently demonstrated the strongest influence on MDC levels, implying their potential as modulators of autophagy. The observed variability among the compounds underscores the importance of structural differences in determining their biological activities.

## 4. Discussion

To investigate novel anticancer therapies, it is crucial to determine their efficacy against cancer cells and assess their selectivity and safety in healthy tissues. This approach is essential for identifying compounds with significant therapeutic potential and minimal adverse effects [28,29]. The literature emphasizes that ideal chemotherapeutic agents should exhibit toxicity against tumor cells while minimally affecting healthy cells, leading to better patient tolerance and reduced systemic side effects [30,31]. Blood cells, such as erythrocytes, platelets, and peripheral blood mononuclear cells (PBMCs), serve as vital models for preliminary toxicity and safety assessments. Their accessibility, importance for oxygen transport, hemostasis, and immune function, along with their sensitivity to external factors, make them effective indicators of adverse drug interactions [32,33]. Studying the impact of chlorochalcones on these cells allows for the prediction of their possible side effects on the circulatory system, which is crucial in the early stages of drug discovery.

To comprehend the biological activity, selectivity, and adverse reactions of a compound, examining its interactions with blood cells is fundamental [34]. As the main cellular constituent of the blood, erythrocytes are an effective model for evaluating the toxicity of novel and established pharmaceutical compounds. The loss of RBC integrity and release of hemoglobin indicate hemolysis, signifying RBC destruction and reduced oxygen transport capacity. Our analysis of the hemolytic activity of 2′-OH chalcones with a chlorine atom revealed that these compounds do not destructively impact erythrocyte membrane integrity. The investigated compounds did not induce hemolysis at concentrations up to two to ten times higher than the half-maximal inhibitory concentrations (IC_50_ values) determined for MDA-MB-231 and MCF-7 cell lines (Table S1). This finding aligns with previous research. Sinha et al. (2019) reported that 18 chalcone derivatives with diverse substitutions on rings A and B showed no adverse effects on erythrocytes at concentrations where antimalarial efficacy was observed against both chloroquine-sensitive and chloroquine-resistant strains [19].

Key factors in maintaining proper RBC function are the physical characteristics of the erythrocyte membrane, including osmotic resistance, transmembrane potential, and cell shape. Erythrocyte osmotic characteristics are clinically significant for substance passage across biological membranes. Osmotic fragility data for mammalian erythrocytes are presented as sigmoidal hemolytic curves, plotting hemolysis percentage against extracellular NaCl concentration (Figure S1) [35]. Quantitatively, osmotic resistance is defined by the C_50_ concentration, representing the NaCl concentration at which 50% of erythrocytes undergo hemolysis (Table 1). For human erythrocytes, this value typically approximates 0.46% [36], a finding that aligns with our own experimental data. At concentrations up to 100 µM, the tested chlorochalcones had no significant effect on the osmotic resistance of erythrocytes, indicating these compounds do not alter the cells’ inherent capacity to respond to osmotic shifts. These observations align with previous research, such as that by Saxena et al. (2007), who found that chalcone derivatives cytotoxic to cancer cell lines did not impair erythrocyte fragility, suggesting that such compounds may be considered nontoxic to healthy cells [20].

The phospholipid bilayer and spectrin network in erythrocytes contribute to their discocyte morphology, providing membrane elasticity and biological characteristics. The shape of erythrocytes can be altered by various physical, chemical, or genetic factors. As posited by Sheetz and Singer [37], the integration of exogenous substances into either membrane monolayer can disturb the equilibrium of the bilayer, inducing alterations in the erythrocyte morphology. Studies have demonstrated that chlorinated derivatives of 2′-hydroxychalcone induce the formation of stomatocytes I and II (Figure 2b), as classified by Bessis [25]. Our previous studies indicated that these compounds exhibit high lipophilicity and interact with model lipid membranes by altering their physical properties [5]. The formation of stomatocytes under the influence of chlorochalcones may be linked to alterations in the membrane lipid bilayer, which changes the membrane curvature. Stomatocyte development is attributed to modifications in membrane fluidity and flexibility and alterations in membrane proteins crucial for cytoskeletal support and membrane integrity [38].

To assess whether the tested compounds influence membrane permeability to ions, studies have evaluated their effect on erythrocyte transmembrane potential using the dual radiometric method with the DiSC_3_(5) probe. The transmembrane potential reflects the potassium permeability of the erythrocyte membrane; for normal human erythrocytes, it typically ranges from −10 to −15 mV [24]. This potential is determined by the concentrations of ions within and outside the cell. The results demonstrated that the chalcones under investigation did not alter the transmembrane potential of erythrocytes (Table 1), indicating that their interaction with the erythrocyte membrane did not influence potassium permeation. These findings align with the osmotic fragility data and suggest that the observed shape changes in erythrocytes resulted from the interaction of compounds with the cell membrane or proteins embedded within it while the membrane osmotic and electrochemical properties remained uncompromised.

The interaction of 2′-hydroxychalcone and chlorine-containing derivatives with erythrocytes in vitro at 100 µM induced alterations in erythrocyte morphology. This occurred without compromising membrane integrity or functional properties with respect to osmotic or electrochemical characteristics. The research demonstrates that the tested chlorochalcones exert no deleterious effect on erythrocytes, even at concentrations exceeding their half-maximal inhibitory concentrations in breast cancer cells.

PLTs and PBMCs are other critical blood components that perform distinct physiological functions. Human PBMCs are identifiable by their spherical nucleus and comprise immune cell types including lymphocytes, monocytes, natural killer (NK) cells, and dendritic cells [39]. Impairment of these fundamental elements of the immune system can result in compromised immune responses. Research has shown that chalcones can modulate PBMC activity with and without mitogenic stimuli. The chemical structures of chalcones determine whether their effects involve toxicity, immune stimulation, or immune inhibition. Rao et al. (2004) [40] demonstrated that certain 2′-oxygenated chalcone derivatives exhibit toxicity towards phytohemagglutinin (PHA)-activated PBMCs. These compounds increased the number of cells in the G2/M phase, correlating with a reduced number of cells in the S phase of the cell cycle [40]. Experiments with normal quiescent and PHA-activated healthy PBMCs showed no toxicity of indole-based chalcones at concentrations up to 10 µM after 24 h exposure [41].

The effects of 2′-hydroxychalcone and its chlorine-substituted derivatives on PBMCs remain unexplored. We investigated the impact of these compounds on normal and quiescent PBMC viability after 24 h of exposure. Cellular viability was assessed based on mitochondrial reductive capacity. The results showed that chalcones had a minimal impact on PBMC viability at concentrations up to 50 µM, a concentration 2–3 times higher than the half-maximal inhibitory concentrations for breast cancer cells. At concentrations of 100 µM or higher, a significant reduction in metabolically active cells was observed, depending on the position and number of chlorine substituents. The compounds most active against PBMCs were those with a single chlorine atom in the B ring.

Platelet aggregation is essential for maintaining hemostasis and may be induced by endogenous agonists such as arachidonic acid (AA), adenosine diphosphate (ADP), thrombin, and collagen [42]. The inhibition of platelet function represents a promising approach for treating thrombotic diseases. Studies have shown that certain chalcones can inhibit platelet aggregation by targeting key pathways involved in thrombosis and inflammation. Licochalcones inhibit collagen-induced platelet aggregation by inhibiting COX-1 activity [43]. A geranylated chalcone from *A. altilis* leaves demonstrated antiplatelet activity by inhibiting ADP-induced platelet aggregation initially and later inducing platelet disaggregation [44]. Lin et al. (1997) reported that dihydroxychalcones exhibited selective inhibitory effects on AA-induced platelet aggregation, with weaker effects on collagen- and thrombin-induced aggregation [45]. These findings prompted us to investigate whether 2′-hydroxychalcone and its chlorinated derivatives inhibit collagen-induced platelet aggregation.

Studies have shown that 2′-hydroxychalcone potently inhibits collagen-induced platelet aggregation. However, the addition of a chlorine atom to its structure significantly reduced its inhibitory potential. Since mitochondrial activity in platelets is directly linked to their functionality through regulation of calcium homeostasis, which is critical for platelet activation and aggregation, we evaluated the effect of these chalcones on PLT mitochondrial metabolic activity. The results indicated that platelets exposed to the studied chalcones for 90 min showed metabolic activity comparable to that of the controls. The exceptions were compounds C1 and C3, which significantly reduced the metabolic activity at concentrations above 50 µM. These compounds also showed a lower ability to inhibit collagen-induced platelet aggregation compared with 2′-hydroxychalcone, suggesting different mechanisms for aggregation inhibition. Further research is needed to elucidate these mechanisms by investigating the signaling pathways and activation processes in platelets.

Our investigations into 2′-hydroxychalcone derivatives with chlorine demonstrated their ability to target cancer cells while sparing normal circulatory system cells. Their high antiproliferative activity against the breast cancer cell lines MDA-MB-231 and MCF-7, shown by IC_50_ values (Table S1) contrasted with their minimal impact on blood component integrity and function. This selectivity, shown by the absence of hemolysis, preservation of erythrocyte osmotic resistance and transmembrane potential, and low toxicity to PBMCs and platelets at therapeutic concentrations, supports its further development as an anticancer drug candidate. These findings show that the studied chlorochalcones, particularly compound C3, represent promising compounds that target malignant cells while minimizing damage to normal cells, which is a key criterion for effective oncological therapies.

Building on these insights into their selective action, the present study further investigated the impact of these compounds on the proliferation of normal HMEC-1. The HMEC-1 cell line retains the morphological, phenotypic, and functional characteristics of normal human microvascular endothelial cells, including cobblestone morphology and the expression of von Willebrand factor and factor VIII. Thus, it serves as a suitable in vitro model for evaluating the potential antitumor effects of various compounds [37]. Our results indicate that the chalcones tested inhibited endothelial cell proliferation to varying degrees, depending on the site of chlorine substitution (Table S1). The most pronounced reduction in cell viability was observed with 2′-hydroxychalcone (C0) and its derivatives containing a chlorine atom at positions 2 and 4 of the B ring (C2 and C4). Moreover, unlike in breast cancer cells, where chlorine substitution enhanced antiproliferative activity, the activity of 2′-hydroxychalcone either remained unchanged or decreased following chlorine substitution in endothelial cells. Among the compounds tested, C3 exhibited the most promising anticancer potential, demonstrating the highest toxicity toward breast cancer cells, while maintaining relatively low toxicity toward normal endothelial cells (Table S1). The lower toxicity of chlorochalcones toward endothelial cells compared with breast cancer cells suggests a degree of selectivity, highlighting their potential as anticancer agents. However, the observed toxicity toward endothelial cells also implies a possible antiangiogenic effect, which may further contribute to their chemopreventive action.

To elucidate the mechanism underlying the cytotoxicity of 2′-hydroxychalcones, we assessed the mode of cell death induced by these compounds in MDA-MB-231 and MCF-7 cells. The ability of the compounds to induce programmed cell death was determined, showing that they triggered apoptosis in both cancer cell lines to varying extents (Figure 5). Comparing their proapoptotic effects indicated a greater ability to modify MCF-7 cells than MDA-MB-231 cells, consistently with previously demonstrated cytotoxicity against these cell lines [23]. It should be noted that apoptosis was assessed at only two representative time points (24 h and 48 h). While these intervals allowed for the observation of early and late apoptotic responses, a more detailed kinetic analysis could provide additional insights into the dynamics of this process. The potential of chalcones to induce apoptosis has been reported by other authors. The ability of 2,3,4′-trimethoxy-2′-hydroxy-chalcone and 3′-bromo-3,4-dimethoxy-chalcone to stimulate human hepatoma cells to initiate programmed cell death was demonstrated by Ramirez-Tagle et al., 2016 [15]. Similarly, 2′-hydroxychalcone and its methoxylated derivatives induce apoptosis in canine lymphoma and leukemia cells [46]. Some chalcones may induce changes in the cell cycle, which is linked to their cell-death-inducing effects. Ibrahim et al. (2024) showed that chalcones possessing 2-phenoxy-N-arylacetamide and thiophene moieties induced apoptosis and cell cycle arrest at the G0/G1 phase in MCF-7 cells [47]. It was also shown that chalcones trigger apoptosis in human bladder cancer cells primarily through cell cycle changes, specifically blocking progression at the G2/M phase [48].

To determine if the apoptosis induced by chlorochalcones is linked to cell cycle regulation, we studied the impact of these compounds on the cell cycle of MCF-7 and MDA-MB-231 cells. The results presented in Figure 6 indicate that the chalcones used practically did not induce significant changes in the cell cycle. The exception was compound C5, which caused a slight cell cycle arrest at the G0/G1 phase in MCF-7 cell line. This suggests that the 2′-hydroxychalcone derivative possessing two chlorine atoms in the A ring may have potential as a therapeutic agent, particularly if the G0/G1 arrest selectively targets cancer cells.

The studied compounds induced apoptosis without significantly affecting cell cycle progression, suggesting that they may directly trigger apoptotic pathways rather than inducing apoptosis indirectly through cell cycle arrest. Apoptosis is a cellular response to a variety of stress factors. The intracellular factors include DNA damage, increased ROS levels, and mitochondrial damage. Using the MTT assay, a significant reduction in mitochondrial enzyme activity was observed in MDA-MB-231 cells treated with 2′-hydroxychalcone and its chloroderivatives. This reduction in metabolic activity may indirectly suggest mitochondrial damage, potentially resulting from elevated levels of ROS. Consequently, we assessed the effect of chalcones on ROS levels in MDA-MB-231 cells using an H_2_DCF-DA tracer (Figure 7a). The results indicated that chlorine-containing derivatives modulated ROS levels in cells, whereas 2′-hydroxychalcone did not induce significant changes compared with control cells. Compound C0 also exhibited the lowest toxicity towards MDA-MB-231 cells. Significant increases in intracellular ROS levels were observed for compounds C2, C4, and C5, which demonstrated the highest antiproliferative activity (Table S1). Interestingly, compound C3 caused a slight decrease in intracellular ROS levels. This compound exhibited approximately half the cytotoxicity towards MDA-MB-231 cells compared with compounds C2 and C4. These data suggest that the presence of a chlorine atom in the 2′-hydroxychalcone structure plays a pivotal role in the modulation of intracellular ROS levels and that the position of chlorine within the B ring significantly influences the ability of the compounds to modulate ROS.

Given the induction of the intrinsic apoptotic pathway following chalcone treatment, mitochondrial functionality was examined by assessing the mitochondrial mass and membrane potential. Among the tested compounds, C3 and C5 induced reductions in MitoTracker Red fluorescence intensity (Figure 7b), indicating mitochondrial membrane depolarization in MDA-MB-231 cells. Furthermore, MitoTracker Green revealed that compounds C0, C2, C3, and C5 significantly reduced mitochondrial content (Figure 7c), as this probe quantifies mitochondrial mass independently of the membrane potential [49]. As main producers of intracellular ROS, mitochondria play a pivotal role in apoptosis by promoting lipid and protein oxidation and DNA damage [50].

**Figure 9:**
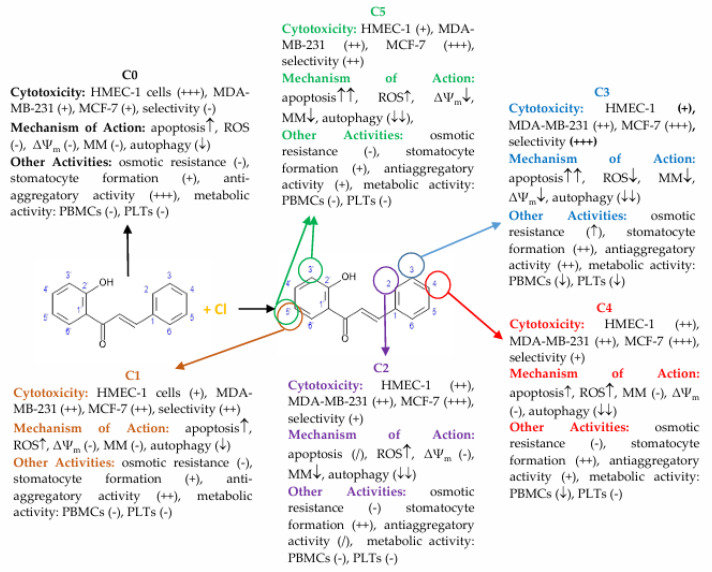
Cytotoxicity, selectivity, mechanisms of action, and additional biological activities of 2′-hydroxychalcone (C0) and its chlorine-containing derivatives (C1–C5). The central panel shows the chemical structure of 2′-hydroxychalcone, with circles marking the carbon atoms in the A and B rings where chlorine substitution occurs. Each derivative is described in terms of its activity profile. Cytotoxicity is shown for HMEC-1 (normal endothelial cells), MDA-MB-231, and MCF-7 (breast cancer cells), and selectivity is defined as the preferential cytotoxicity towards cancer cells. Mechanisms of action include induction of apoptosis, modulation of reactive oxygen species (ROS), changes in mitochondrial membrane potential (ΔΨ_m_) and mitochondrial mass (MM), and effects on autophagy. Additional activities are presented for blood cells, including effects on osmotic resistance, erythrocyte shape alterations (stomatocyte formation), antiaggregatory properties (collagen-induced platelet aggregation), and changes in the metabolic activity of PBMCs and platelets (PLTs). Symbols denote intensity and direction of effects: “+” = low, “++” = moderate, “+++” = strong; arrows indicate increase (↑) or decrease (↓) in the parameter, with multiple arrows reflecting stronger effects; “-” = no effect observed; “/” = parameter not determined.

The increase in intracellular ROS levels and mitochondrial dysfunction suggests chlorine-substituted 2′-hydroxychalcones may induce apoptosis in breast cancer cells via the intrinsic pathway. Previous studies have shown that chalcones influence the mitochondrial function. The chalcone ETTC [(E)-1-(2,4,6-triethoxyphenyl)-3-(3,4,5-trimethoxyphenyl)prop-2-en-1-one] decreased mitochondrial membrane potential and mass in HepG2 cancer cells [51]. Similarly, cyclic chalcones, E-2-(4′-methoxybenzylidene)-1-benzosuberone and E-2-(4′-dimethylaminobenzylidene)-1-benzosuberone, caused mitochondrial dysfunction and DNA damage in rat liver cells [52]. Our findings show that the chlorochalcone derivatives C2 and C5 affected the mitochondrial membrane potential more than other derivatives, causing dose-dependent mitochondrial depolarization. This loss of mitochondrial membrane potential (MMP) indicates dysfunction and correlates with increased phosphatidylserine externalization observed via Annexin V staining. These results suggest C2 and C5 induce apoptosis through mitochondrial pathways, with depolarization as a key upstream event. C0, C2, C3, and C5 showed reduced mitochondrial mass at higher concentrations, suggesting activation of mitochondrial clearance mechanisms like mitophagy. This aligns with reports that chalcone derivatives promote mitophagy during cellular stress [11,53]. The sequence of mitochondrial depolarization followed by reduced mitochondrial content indicates that dysfunction precedes organelle removal. These findings match studies on other chalcone derivatives. Isoliquiritigenin decreased MMP and induced apoptosis in breast cancer cells by modulating Bcl-2 proteins and activating caspase cascades [54]. Chalcone-based hybrids such as protoflavones can depolarize mitochondrial membranes and promote mitophagy in cancer models [55]. Although C2 and C5 primarily induce apoptosis, they may act through distinct mitochondrial pathways compared with C3 and C4, which drive mitophagy. This dual mechanism has been observed for other mitochondrial-targeting compounds [56]. Our results showed that chlorochalcone derivatives affected mitochondrial function. C2 and C5 disrupted membrane potential to induce apoptosis, while C2, C3, and C5 promoted mitochondrial degradation through mitophagy. A comprehensive summary of these findings, illustrating the relationship between 2′-hydroxychalcone and its chlorine-substituted derivatives with respect to their diverse biological activities, is provided in Figure 9. These findings highlight chlorochalcone derivatives as promising anticancer therapeutic candidates warranting further investigation.

## 5. Conclusions

This study provides a comprehensive evaluation of the anticancer activity and safety profile of chlorochalcones, with particular emphasis on their effects on breast cancer cells, blood components, and endothelial cells. Chlorochalcones exhibited potent antiproliferative effects against breast cancer cell lines while showing minimal toxicity toward erythrocytes, PBMCs, and platelets at therapeutic concentrations. Importantly, their lower toxicity toward endothelial cells compared with breast cancer cells indicates a degree of selectivity and suggests potential antiangiogenic activity.

The results provide mechanistic insights, suggesting that chlorochalcones act mainly through the intrinsic mitochondrial pathway, with accompanying effects on ROS modulation, mitochondrial membrane depolarization, and mitochondrial mass reduction. Their ability to influence autophagy and mitophagy further highlights the complexity of their action, with different derivatives engaging distinct cell death mechanisms.

The structure–activity relationship analysis demonstrated that the position and number of chlorine atoms play a decisive role in biological activity. Among the tested compounds, C3 emerged as the most promising candidate, combining strong cytotoxicity toward breast cancer cells with relatively low toxicity to normal endothelial cells.

This study underscores the importance of comprehensive safety profiling during the early stages of drug discovery and provides a foundation for future research aimed at developing more effective and safer anticancer therapies.

## Figures and Tables

**Table 1:** The NaCl concentration (%) at which 50% hemolysis of the control and chalcone-modified erythrocytes occurred. Osmotic fragility data were fitted to a sigmoid curve to calculate C_50_. The transmembrane potentials (ΔΨ) of control and chalcone-modified erythrocytes are also presented. The results are presented as mean values from at least three independent experiments ± standard deviation.

Compound/Concentration	C_50_, %		ΔΨ, mV
	40 µM	100 µM	100 µM
Control	0.471 ± 0.014	0.465 ± 0.012	−10.8 ± 1.4
C0	0.467 ± 0.013	0.465 ± 0.007	−11.5 ± 0.9
C1	0.454 ± 0.004	0.460 ± 0.009	−11.4 ± 1.2
C2	0.469 ± 0.008	0.469 ± 0.007	−11.4 ± 0.4
C3	0.470 ± 0.011	0.449 ± 0.004 *	−12.6 ± 1.3
C4	0.461 ± 0.015	0.445 ± 0.021	−10.6 ± 1.2
C5	0.469 ± 0.012	0.469 ± 0.038	−10.4 ± 0.4

* Statistically significant differences, α = 0.05.

## Data Availability

The data presented in this study are openly available in Base of Knowledge, Wrocław University of Environmental and Life Sciences, DOI:10.57755/f97g-5j91.

## Supplementary Materials

The following supporting information can be downloaded at: https://www.mdpi.com/article/10.3390/cells14161299/s1, Figure S1: Osmotic resistance curves of control erythrocytes and those modified with chalcones at 40 µM (a) and 100 µM (b). Chlorinated derivatives of 2′-hydroxychalcone (C0): 5′-chloro-2′-hydroxychalcone (C1), 2-chloro-2′-hydroxychalcone (C2), 3-chloro-2′-hydroxychalcone (C3), 4-chloro-2′-hydroxychalcone (C4), and 3’,5’-dichloro-2’-hydroxychalcone (C5). The compounds were incubated with RBCs for 1 h at 37 °C. Statistically significant differences between control and chalcone-modified erythrocytes are expressed as * *p* < 0.01; Table S1: The antiproliferative effects of 2’-hydroxychalcone and its chlorine derivatives in relation to HMEC-1 cells. The modification of HMEC-1 cells lasted 72 h. The IC50 values are presented as mean ± SD of three independent experiments. Chlorinated derivatives of 2’-hydroxychalcone (C0): 5’-chloro-2’-hydroxychalcone (C1), 2-chloro-2’-hydroxychalcone (C2), 3-chloro-2’-hydroxychalcone (C3), 4-chloro-2’-hydroxychalcone (C4), and 3’,5’-dichloro-2’-hydroxychalcone (C5). Doxorubicin (DOX) was treated as a standard; Figure S2: Example images of control erythrocytes (a) and those treated with chlorochalcones C0 (b), C3 (c), and C5 (d) used at a concentration of 100 μM; Figure S3: Representative Annexin V–FITC/propidium iodide dot plots illustrate apoptosis in MCF-7 (a) and MDA-MB-231 (b) cells following 24-h and 48-h exposure to compound C0, C3, or C5 (40 µM), in comparison with the untreated control. The percentages of live (Q4), early apoptotic (Q3), late apoptotic/dead (Q2), and necrotic (Q1) cell populations are shown. Figure S4: Representative cell-cycle histograms of propidium iodide–stained MCF-7 (a) and MDA-MB-231 (b) cells collected 24 h after treatment with chlorochalcones. DNA content (PI-A, x-axis) was plotted against cell count (*y*-axis) from left to right for the untreated control, C0 (40 µM), and C3 or C5 (40 µM). Colored curve fits generated using the Watson pragmatic model resolved the G0/G1 (green), S (tan), and G2/M (blue) phases. Corresponding phase frequencies and model statistics are provided in the in-panel tables labelled as control, C0, and C3 or C5.
