# Supplementary material for: Selective Anticancer Activity and Safety Profile of Chlorochalcones: Impact on Breast Cancer, Blood, and Endothelial Cells
Source: Cells. 2025 Aug 21;14(16):1299. doi: 10.3390/cells14161299 (PMC12384567; doi:10.3390/cells14161299)
Supplement: Supplementary file 1 [file cells-14-01299-s001.zip › cells-3791488-supplementary.pdf]

Supplementary materials

Selective Anticancer Activity and Safety Profile of Chlorochalcones: Impact on Breast Cancer, Blood, and Endothelial Cells

Sylwia Cyboran-Mikołajczyk [1,*], Karolina Matczak [2], Teresa Kaźmierczak [1], Natalia Trochanowska-Pauk [1], Tomasz Walski [3], Raghvendra Bohara [4], Karol Bukowski [2], Agnieszka Krawczyk-Łebek [5] and Edyta Kostrzewa-Susłow [5]

[1] Department of Physics and Biophysics, Faculty of Biotechnology and Food Sciences, Wrocław University of Environmental and Life Sciences, Norwida 25 St., 50-375 Wrocław, Poland; teresa.kazmierczak@upwr.edu.pl (T.K.); natalia.trochanowska-pauk@upwr.edu.pl (N.T.-P.)

[2] Department of Medical Biophysics, Faculty of Biology and Environmental Protection, University of Lodz, Pomorska 141/143 St., 90-236 Lodz, Poland; karolina.matczak@biol.uni.lodz.pl (K.M.); karol.bukowski@biol.uni.lodz.pl (K.B.)

[3] Department of Biomedical Engineering, Faculty of Fundamental Problems of Technology, Wrocław University of Science and Technology, Wybrzeże Wyspiańskiego 27, 50-370 Wrocław, Poland; tomasz.walski@pwr.edu.pl

[4] Centre for Interdisciplinary Research, D.Y. Patil Educational Society, Kolhapur 416006, India; raghvendrabohara@gmail.com

[5] Department of Food Chemistry and Biocatalysis, Wrocław University of Environmental and Life Sciences, Norwida 25 St., 50-375 Wrocław, Poland; agnieszka.krawczyk-lebek@upwr.edu.pl (A.K.-Ł.); edyta.kostrzewa-suslow@upwr.edu.pl (E.K.-S.)

* Correspondence: sylwia.cyboran@upwr.edu.pl

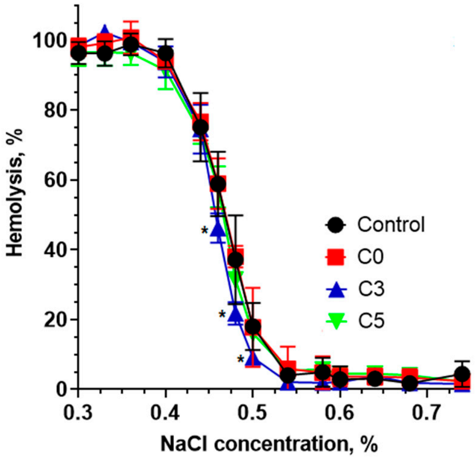

a) b)

Figure S1. Osmotic resistance curves of control erythrocytes and those modified with chalcones at 40 µM (a) and 100 µM (b). Chlorinated derivatives of 2'-hydroxychalcone (C0): 5'-chloro-2'-hydroxychalcone (C1), 2-chloro-2'-hydroxychalcone (C2), 3-chloro-2'-

hydroxychalcone (C3), 4-chloro-2'-hydroxychalcone (C4), and 3',5'-dichloro-2'-hydroxychalcone (C5). The compounds were incubated with RBCs for 1 h at 37 °C. Statistically significant differences between control and chalcone-modified erythrocytes are expressed as $^{*}p < 0.01$.

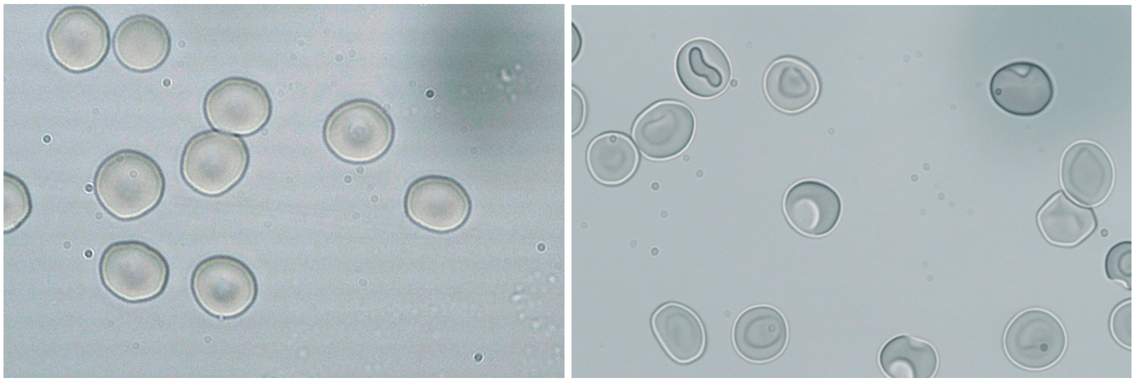

a) b)

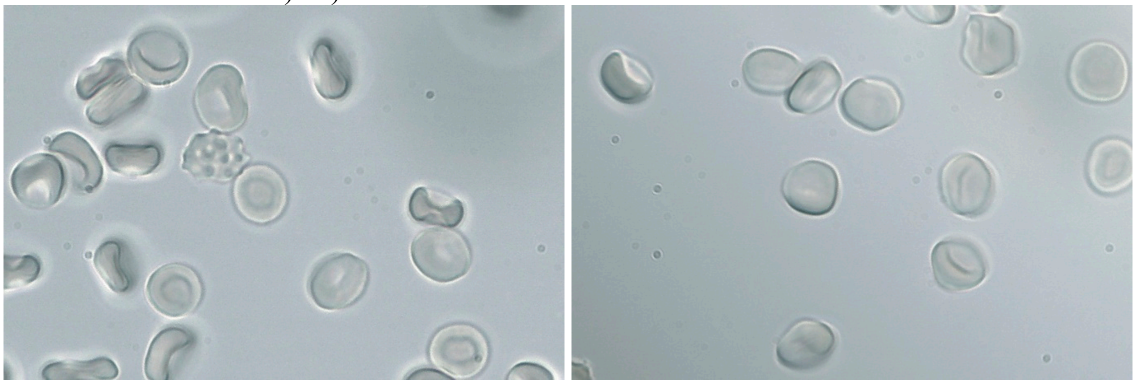

c) d)

Figure S2. Example images of control erythrocytes (a) and those treated with chlorochalcones C0 (b), C3 (c), and C5 (d) used at a concentration of 100 μM.

Table S1. Antiproliferative effects of 2’-hydroxychalcone and its chlorine derivatives on HMEC-1 cells. The modification of the HMEC-1 cells lasted for 72h. The IC50 values are presented as the mean ± SD of three independent experiments. Chlorinated derivatives of 2'-hydroxychalcone (C0): 5'-chloro-2'-hydroxychalcone (C1), 2-chloro-2'-hydroxychalcone (C2), 3-chloro-2'-hydroxychalcone (C3), 4-chloro-2'-hydroxychalcone (C4), and 3',5'-dichloro-2'-hydroxychalcone (C5). (DOX) was used to treat the cells as the standard.

**Compound**	**HMEC-1**	**MDA-MB-231***	**MCF-7***
		**IC_{50} [μM]**	
**Time**		**72h**	
C0	17.9 ± 0.5	75.5	61.0
C1	51.5 ± 2.6	33	31.4
C2	16.8 ± 0.4	17.1	6.0
C3	63.9 ± 2.2	20.3	6.1
C4	15.3 ± 0.7	12.9	4.3
C5	38.3 ± 0.9	12.5	6.3
**DOX**	**0.08 ± 0.013**	**0.45**	**0.12**

* results were published earlier in [6].

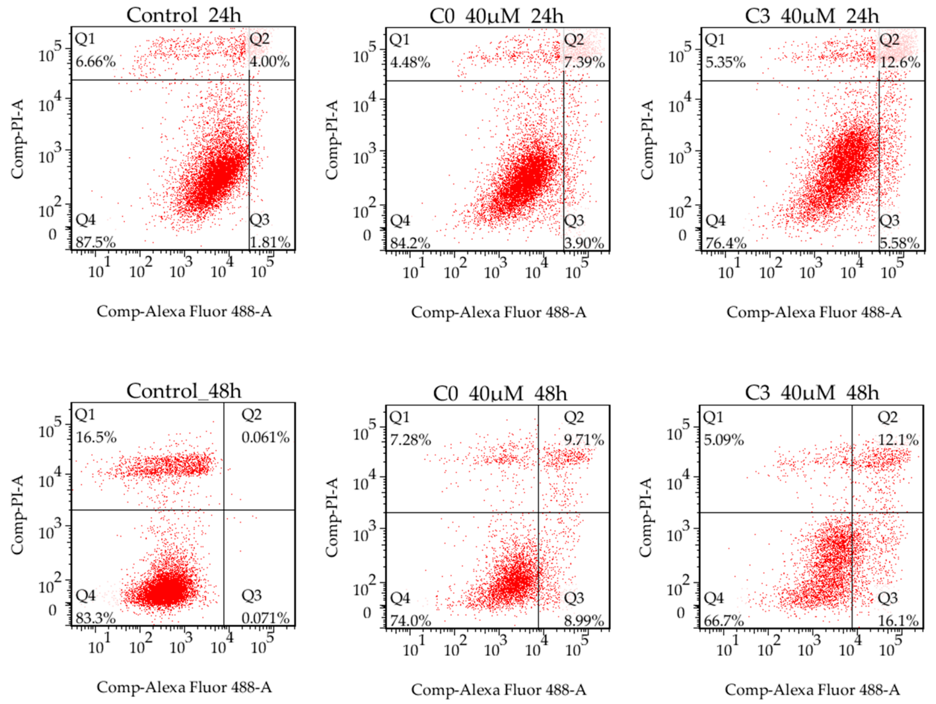

a)

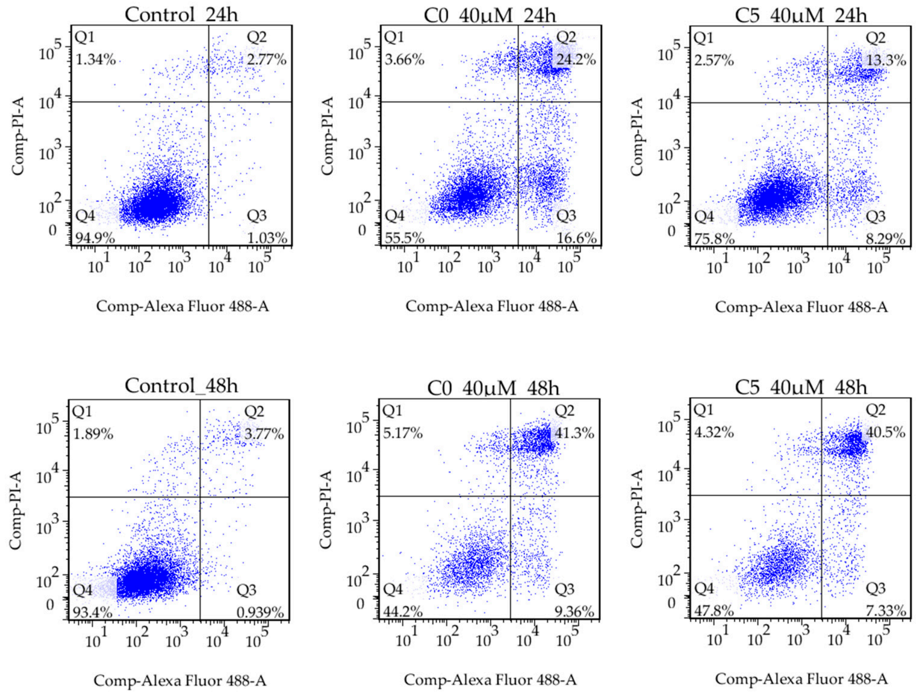

b)

Figure S3. Representative Annexin V–FITC/propidium iodide dot plots illustrate apoptosis in MCF-7 (a) and MDA-MB-231 (b) cells following 24-hour and 48-hour exposure to compounds C0, C3, or C5 (40 μM), in comparison to the untreated control. The percentages of live (Q4), early apoptotic (Q3), late apoptotic/dead (Q2), and necrotic (Q1) cell populations are shown.

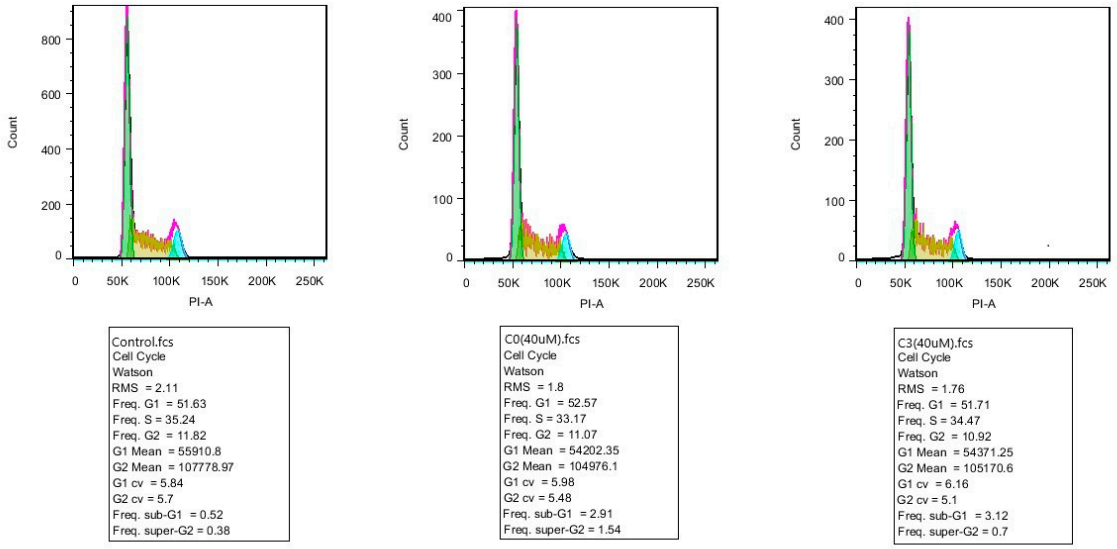

a)

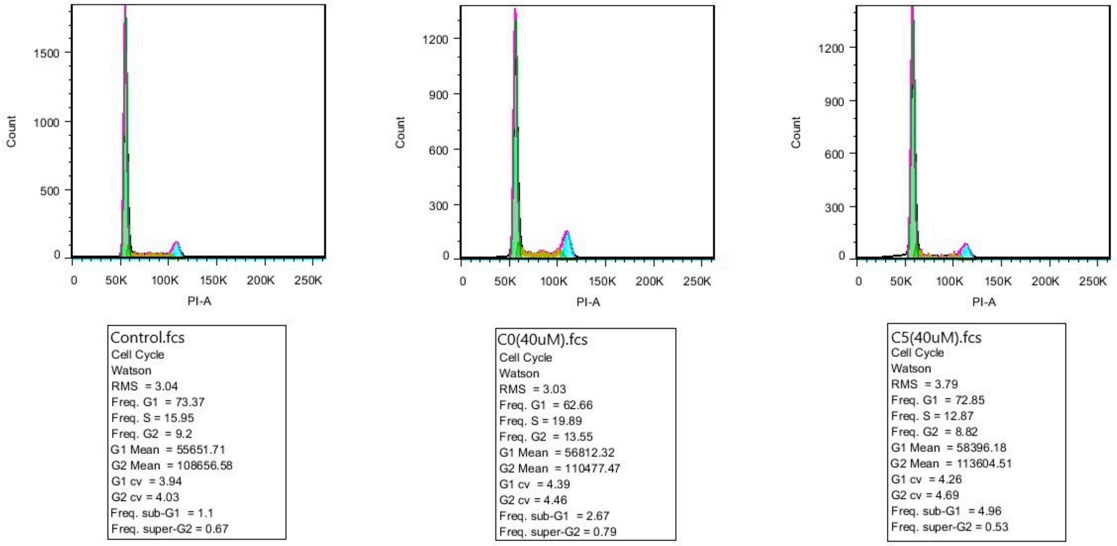

b)

Figure S4. Representative cell-cycle histograms of propidium iodide–stained MCF-7 (a) and MDA-MB-231 (b) cells collected 24 h after treatment with chlorochalcones. DNA content (PI-A, x-axis) was plotted against cell count (y-axis) from left to right for the untreated control, C0 (40 µM), and C3 or C5 (40 µM). Colored curve fits generated using the Watson pragmatic model resolved the G0/G1 (green), S (tan), and G2/M (blue) phases. Corresponding phase frequencies and model statistics are provided in the in-panel tables labelled as control, C0, and C3 or C5, respectively.
